# Influence of Haem, Non-Haem, and Total Iron Intake on Metabolic Syndrome and Its Components: A Population-Based Study

**DOI:** 10.3390/nu10030314

**Published:** 2018-03-07

**Authors:** Diva Aliete dos Santos Vieira, Cristiane Hermes Sales, Chester Luiz Galvão Cesar, Dirce Maria Marchioni, Regina Mara Fisberg

**Affiliations:** 1 Department of Nutrition, School of Public Health, University of Sao Paulo, Av. Dr. Arnaldo, 715, Cerqueira César, São Paulo, SP 01246-904, Brazil; diva.nutricao@gmail.com (D.A.d.S.V.); cristianehermes@yahoo.com.br (C.H.S.); marchioni@usp.br (D.M.M.); 2 Department of Epidemiology, School of Public Health, University of Sao Paulo, Av. Dr. Arnaldo, 715, Cerqueira César, São Paulo, SP 01246-904, Brazil; clcesar@usp.br

**Keywords:** iron intake, epidemiologic surveys, nutritional assessment, food intake, metabolic syndrome

## Abstract

Studies suggest that haem, non-haem iron and total iron intake may be related to non-communicable diseases, especially metabolic syndrome. This study was undertaken to investigate the association of haem, non-haem iron and total iron intake with metabolic syndrome and its components. A cross-sectional population-based survey was performed in 2008, enrolling 591 adults and elderly adults living in São Paulo, Brazil. Dietary intake was measured by two 24 h dietary recalls. Metabolic syndrome was defined as the presence of at least three of the following: hypertension, hyperglycaemia, dyslipidaemia and central obesity. The association between different types of dietary iron and metabolic syndrome was evaluated using multiple logistic regression. After adjustment for potential confounders, a higher haem iron intake was positively associated with metabolic syndrome and with elevated triglyceride levels. A higher total iron intake was positively associated with hyperglycaemia. Non-haem iron intake was positively associated with hyperglycaemia in the fourth quintile. In conclusion, this study suggests that the different types of dietary iron are associated with metabolic syndrome, elevated triglyceride levels and hyperglycaemia. In addition, it emphasises the importance of investigating the roles of dietary iron in health outcomes, since its consumption may have different impacts on health.

## 1. Introduction

Studies indicate that consumption of haem and non-haem iron may be related to the risk of developing metabolic syndrome (MetS), hyperglycaemia and alterations in lipid metabolism [1,2,3]. MetS is a set of risk factors that are interrelated in the pathogenesis of cardiovascular disease and type 2 diabetes [4], which are major causes of morbidity and mortality worldwide [5].

Since these diseases are preventable and diet is among their predisposing factors, identification of nutritional factors that may be associated with an increased risk of developing MetS is relevant to public health. Iron overload has been linked to the formation of reactive oxygen species, especially via the Fenton reaction, that may lead to DNA damage and lipid peroxidation. These conditions have been related to the development of cardiovascular diseases, cancers, and type 2 diabetes [6,7].

Haem and non-haem iron present differences in absorption, metabolism, bioavailability and food sources. Mechanisms that regulate haem and non-haem iron homeostasis are essential for preventing its cytotoxicity. Absorption of dietary iron is regulated by cellular and systemic factors to maintain adequate iron levels [8]. Hepcidin, main regulator of extracellular iron homeostasis, acts as a negative regulator of iron absorption by triggering ferroportin internalization and degradation, limiting the entry of iron into the plasma [9,10]. Mutations in regulatory molecules, such as the hemochromatosis gene, transferrin receptor 2 and hemojuvelin, may cause hepcidin deficiency, resulting in iron overload and mutations in transmembrane protease serine 6, which are associated with severe iron deficiency due to hepcidin excess [11]. The investigation of these different types of iron in MetS may help to clarify their roles in shaping different health outcomes. To our knowledge, no published study has examined the influence of haem and non-haem iron, separately, on MetS and its components. Therefore, the aim of this study was to investigate the association of dietary haem, non-haem iron and total iron intake with MetS and its components.

## 2. Materials and Methods

### 2.1. Study Population

A cross-sectional population-based survey was conducted in São Paulo, Brazil: 2008 Health Survey of São Paulo (“Inquérito de Saúde de São Paulo”, ISA-Capital). In this survey, information about the health, nutrition, and living conditions of a section of the population was collected.

The sample was recruited in two stages: (1) 70 census tracts were randomly selected from 267 urban census tracts in the city of São Paulo (primary sampling); (2) households were randomly selected within these census tracts, considering different probabilities of selection for each domain: (1) infants (<1 year old; both genders); (2) children (1–11 years; both genders); (3) male adolescents (12–19 years); (4) female adolescents (12–19 years); (5) male adults (20–59 years); (6) female adults (20–59 years); (7) male elderly (≥60 years); and (8) female elderly (≥60 years). A sample size of 300 in each domain was estimated to be the minimum, based on a prevalence of 0.5 with a standard error of 0.07 at a 5% significance level and a design effect of 1.5.

A total of 3271 individuals participated in the survey. Of these, 2691 individuals, aged 12 years or over, were selected to answer questions about diet, lifestyle characteristics, and sociodemographic information. Only 1662 individuals of the initial sample agreed to participate. Of those, a subsample of 750 subjects provided a blood sample for biochemical analysis, completed 24 h dietary recalls (24HR), and supplied anthropometric data, as well as arterial blood pressure measurements. For the present study, only adults and elderly adults were included, totalling a final sample of 591 individuals: 301 adults and 290 elderly adults.

The Ethics Committee of the Public Health School of the University of São Paulo approved this survey (protocol # 26800414.1.0000.5421). Written informed consent was obtained from all individuals.

### 2.2. Dietary Assessment

Two 24HR were collected from each individual by trained interviewers. The first was collected in the home, using the Multiple-Pass Method [12], and the second, over telephone, using the Automated Multiple-Pass Method [13]. Both methods follow the same protocols for data collection. The respondent is guided through five steps: (1) a quick list, in which the respondent lists all of the food and beverages consumed in the day before the interview, uninterruptedly; (2) a forgotten list, in which the respondent is asked about commonly consumed forgotten foods, such as alcoholic and non-alcoholic beverages, snacks, fruits, vegetables and candies; (3) time and occasion, information about the time and name of each meal are collected, and the foods and beverages listed above are arranged chronologically and grouped according to the meal; (4) detailing cycle, in which the respondent is asked for detailed descriptions of each food and beverage consumed as well as the brand name, preparation form, type of food and portion consumed; and (5) a final review, in which all food and beverages consumed as well as the time and meals are reviewed and there is a final opportunity to remember and confirm the food and beverages consumed on the previous day. These methods, which uses a standardized process that keeps individuals interested and engaged in the interview, helps them to remember all items consumed [12,13]. All interviewers had an album containing images of commonly utensils to improve the accuracy of the data collected, and, in case of doubt, the interviewee was asked to show the used utensil. The 24HR were collected on non-consecutive days, regardless of day of the week and season.

The information generated from the 24HR was used to assess intakes of iron and other nutrients. The nutrients were estimated using Nutrition Data System for Research software, version 2007 (NDSR, Nutrition Coordinating Center, University of Minnesota, Minneapolis, MN, USA). This software has the United States Department of Agriculture Food Composition Table as its primary database [14]. The nutritional values of the food, as derived from the NDSR, were compared with those in the Brazilian Food Composition table, and the iron content was corrected where it showed differences. The values were corrected through routines created in Stata Statistical Software, version 13 (StataCorp LP, College Station, TX, USA). In Brazil, since 2004, the addition of 4.2 mg of iron per 100 g of flour in maize and wheat flour has been mandatory; thus, a correction was included for the amount of iron added to fortified products. Haem iron was estimated as 40% of the total iron from beef, fish and poultry [15], and non-haem iron was calculated as the difference between total iron and haem iron.

### 2.3. Metabolic Syndrome Assessment

During the study, a trained nursing technician collected 12 h fasting blood samples and recorded blood pressure and anthropometric measurements of the individuals in the household, following standardised protocols.

Fasting plasma glucose levels were analysed using the enzymatic method of glucose oxidase (Glucose Liquiform, Labtest, Lagoa Santa, Minas Gerais, Brazil) using an automated system (LabMax 240, Lagoa Santa, Minas Gerais, Brazil). Plasma triglyceride and high-density lipoprotein cholesterol (HDL-c) levels were analysed with the Roche Modular Auto Analyzer, using the colorimetric-enzymatic method (Roche Diagnostics GmbH, Mannheim, Germany).

Blood pressure measurements were made using a manual oscillometer (Omron^®^, model HEM-712 C, Omron Health Care, Inc., Lake Forest, IL, USA), following the Fifth Brazilian Guidelines for Hypertension [16]. Waist circumference (WC) was measured in duplicate using a flexible, inelastic tape measure, positioned midway between the last rib and the iliac crest.

MetS was identified by the presence of at least three of the following metabolic abnormalities: (1) increased waist circumference (WC ≥ 90 cm for men and WC ≥ 80 cm for women); (2) elevated triglyceride levels (triglycerides ≥ 150 mg/dL) or use of hypolipidaemic drugs; (3) reduced HDL-c levels (HDL-c < 40 mg/dL for men and <50 mg/dL for women) or use of hypolipidaemic drugs; (4) high blood pressure (systolic blood pressure  ≥ 130 mmHg and/or diastolic blood pressure  ≥ 85 mmHg) or use of antihypertensive drugs; and (5) high fasting glucose levels (≥100 mg/dL) or use of hypoglycaemic drugs [4].

### 2.4. Other Covariates

A structured questionnaire was used to gather information on socio-demographics (gender, age, self-reported skin colour, household per capita income), and lifestyle characteristics (smoking habits, alcohol consumption and physical activity). Household per capita income was calculated by summing the monetary income reported and dividing by the number of all family members (R$1.00 = US$1.83, in 2008). Smoking status was ascertained from questions about current or past smoking and the number of cigarettes smoked per day. Alcohol consumption was assessed from information about amount, frequency and preferences. The International Physical Activity Questionnaire long form was used to collect data on physical activity [17], and information from the leisure domain of the questionnaire was used to classify the level of physical activity. Leisure time physical activity was classified as insufficiently active or sufficiently active (physical activity practiced for at least 30 min daily, 5 days per week, at a moderate intensity, or at least 20 min daily, 3 days per week, at a vigorous intensity). The Quetelet equation was used for body mass index (BMI) calculation (BMI = weight (kg)/height (m)^2^), from data measured by trained nurses in the home. The C-reactive protein (CRP) level was measured by kinetic turbidimetry, using an IMMAGE^®^ immunochemistry system kit (Beckman Coulter Inc., Brea, CA, USA). The misreporting percentage of each individual’s energy needs was determined as Total energy intake—EER (estimated energy requirements)/EER × 100 [18]. These variables were used as potential confounders in the analytical models.

### 2.5. Statistical Analysis

Subject characteristics are presented as medians and interquartile ranges (IQR) for continuous variables and as percentages for categorical variables. The nutrients were adjusted for total energy intake using a nutrient residual model [19], and usual intake was estimated using the Multiple Source Method (MSM) [20]. This web-based tool provides usual nutrient intake distributions by removing intrapersonal variability in three steps. The first step estimates the probability of nutrient intake for each individual with a logistic regression model. Secondly, a linear regression model estimates the usual nutrient intake on days of consumption. Finally, the numbers resulting from step one and two are multiplied to estimate the individual usual intake for each individual [21].

Dietary intakes of haem, non-haem iron, and total iron were classified into quintiles. The associations between the quintile categories of haem, non-haem iron and total iron intakes with MetS and its components were analysed using logistic regression models. The analyses were adjusted for physical activity, gender, alcohol consumption, household per capita income, BMI, high-sensitivity C-reactive protein, age, smoking status, race, misreporting, and intakes of total energy, saturated fat, fibre and vitamin C. In logistic regression models where haem iron was a predictive variable, non-haem iron was included in the model as an adjustment variable. The opposite happened when non-haem iron was used as a predictor variable. The Hosmer–Lemeshow goodness-of-fit test was used to assess model fit. All analyses considered the complexity of the sample design and were performed with Stata Statistical Software, version 13 (StataCorp LP, College Station, TX, USA).

## 3. Results

The sample consisted of 591 individuals of whom 80.80% were adults, 53.92% were women, 60.88% self-reported their skin colour as white, 58.85% self-reported never having smoked, 51.88% were alcohol consumers, and 88.52% had insufficient leisure time physical activity. The median household per capita income was 274.87 (IQR 159.03–471.20) US$ per month and the median BMI was 25.61 (IQR 22.96–29.53) kg/m^2^.

Thirty-two percent of these individuals were classified with MetS. Considering the components of MetS separately, 66.02% of the individuals had increased WC, 41.54% reduced HDL-c, 31.01% elevated triglyceride levels, 43.30% had hypertension, and 11.23% had hyperglycaemia. Of these, 5.39% were treated with hypolipidemic, 4.34% with hypoglycaemic and 16.79% with antihypertensive drugs.

In crude models, higher intakes of haem iron (fifth quintile) (odds ratio (OR) = 2.13, 95% confidence interval (95% CI) = 1.01–4.52) were positively associated with MetS, and there was a negative association between the fourth quintiles of total iron intake (OR = 0.57, 95% CI = 0.32–0.99) and MetS. After further adjustment for potential confounders, only higher haem iron intakes (OR = 2.39, 95% CI = 1.10–5.21) were found to be positively associated with MetS when compared to the first quintile (Table 1).

Considering the components of MetS separately, crude models showed positive associations between the fifth quintile of total iron intake (OR = 4.59, 95% CI = 1.71–12.29) and the fourth quintile of nonhaem iron intake (OR = 2.86, 95% CI = 1.19-6.89) with hyperglycaemia, and between the fifth quintile of haem iron intake (OR = 2.73, 95% CI = 1.25–5.96) and elevated triglyceride levels. After further adjustment for potential confounders, higher haem iron intakes (OR = 2.51, 95% CI = 1.06–5.91) were positively associated with elevated triglyceride levels. A higher total iron intake (OR = 3.98, 95% CI = 1.21–13.12) was positively associated with hyperglycaemia. Non-haem iron intake (OR = 2.92, 95% CI = 1.10–7.72) was positively associated with hyperglycaemia in the fourth quintile (Table 2).

## 4. Discussion

To our knowledge, this is the first population-based study examining associations between haem, non-haem iron, and total iron intakes and MetS and its components. Our results suggest that haem iron intake is positively associated with MetS and with elevated triglyceride levels, and non-haem iron and total iron intakes are positively associated with hyperglycaemia.

Epidemiological studies have associated haem iron intake with cardiovascular diseases [22], MetS [2] and type 2 diabetes [23]. However, the aetiology of the association is not well understood. Haem iron has a higher bioavailability compared to non-haem iron, and its main source is dietary red meat. Haem is an important prosthetic group present in many proteins which are essential for life. However, it can be harmful in excess due to its high toxicity [24,25].

de Oliveira Otto et al. [2], when investigating associations of iron and other micronutrients with the incidence of MetS in the Multi-Ethnic Study of Atherosclerosis, found that intakes of haem and haem iron from red meat were positively associated with the risk of MetS—results similar to those found in this study. Higher haem iron intake may lead to overload, and its excess may promote lipid peroxidation and reactive oxygen species and stimulate the production of inflammatory mediators [20]—conditions associated with MetS [26].

The mechanisms underlying the relationship between haem iron intake and elevated triglyceride levels are unclear. However, we hypothesize that higher haem iron intake might increase the risk of haem overload, which can promote generation of free radicals and inflammation [25], which are causally involved in insulin resistance. In hyperinsulinaemia or insulin resistance states, there may be reduced insulin-mediated suppression of hormone-sensitive lipase, an enzyme responsible for triglyceride mobilization. In these conditions, intracellular lipolysis is increased, leading to elevated circulating levels of free fatty acids and their increased transport to the liver. This increase in the levels of hepatic free fatty acids stimulates the production of triglyceride-rich lipoprotein and very low-density lipoprotein in the liver and in intestinal chylomicrons [27,28,29]. Thus, our findings are consistent with previous studies that showed an association between red meat intake (the main source of haem iron) and elevated triglyceride levels [30,31,32].

A positive association between hyperglycaemia and non-haem iron was observed in the second and fourth quintiles. Individuals classified into these consumption quintiles showed lower bean intakes when compared to other quintiles (data not shown). Non-haem iron is present in different foods from plant and animal sources. In Brazil, after the mandatory iron fortification policy was implemented in 2004, wheat and maize flour became important vehicles for iron. As non-haem iron is present in foods that may be beneficial for the prevention and treatment of non-communicable diseases, such as dark green leafy vegetables and beans, the effect of this nutrient may be attenuated by the presence of other components in these foods.

Several studies have shown the effect of the consumption of beans on the glycaemic response in different populations [28]. Possible explanations for the protective role of beans in the glycaemic response are the high contents of fibre and protein, low glycaemic index, high amylose starch and antinutrients. These factors, singly or in association with others, act by delaying gastric emptying, thus reducing the glycaemic response [33]. Therefore, a greater consumption of beans is probably protective against the deleterious effects of iron on the glycaemic response.

An association between total iron intake and the increased risk of hyperglycaemia has been found in a prospective study and in a cross-sectional household survey [1,34]. According to Fernández-Real, McClain and Manco [35], iron overload promotes changes in glucose metabolism, causing hyperinsulinaemia, either by decreased extraction of insulin or by impaired insulin signaling. Choi et al. [3] observed that mice on a high-iron diet showed hyperglycaemia, hyperinsulinaemia and insulin resistance, as well as harmful effects on fatty acid and glucose metabolism. Besides this, despite not being assessed in the present study, it has been demonstrated that changes in normal ranges of ferritin and transferrin saturation can influence diabetes and cardiovascular risks [36].

The different effects of haem and non-haem iron that we found in this study can be attributed to differences in metabolism, bioavailability and food source. In addition, there is evidence in the literature that there are differences in metabolic vulnerability to excess iron according to racial health disparities [37]. The present study has some limitations that should be considered. The cross-sectional design does not permit inferences of causality between exposures and outcomes. Despite adjusting for known confounders, residual confounding cannot be excluded from our findings. Another limitation of the study is inherent to the dietary intake evaluation methods; however, care was taken to minimize biases, such as by applying two 24HR, using the MSM to estimate usual intake, and correcting iron intake data from the Brazilian Food Composition table. The absence of models that estimate the iron haem content of meat that considers the specificities of Brazil is also another limitation of the study. The use of another method for estimating the haem iron content may lead to different results for the present study. The use of the Monsen model allows us to compare our study with other similar studies.

## 5. Conclusions

This study suggests that a higher haem iron intake is positively associated with MetS and elevated triglyceride levels, while higher non-haem and total iron intakes are positively associated with hyperglycaemia. In addition, this study stresses the importance of investigating the different types of dietary iron in relation to health outcomes, since their consumption may have different impacts on health.

## Figures and Tables

**Table 1:** Association between quintiles of iron intake (haem, non-haem and total) and metabolic syndrome in ISA-Capital. São Paulo, Brazil, 2008.

Models	Quintiles of Iron Intake by Class, Odds Ratio (95% Confidence Interval)					*p* for Trend
	Q1	Q2	Q3	Q4	Q5	
**Haem Iron**						
Dietary intake (mg/day)	0.56	0.74	0.91	1.12	1.40	
Crude	1.00	1.74 (0.92–3.29)	1.35(0.69–2.64)	0.88 (0.40–1.93)	2.13 (1.01–4.52)	0.375
Model adjusted ^a^	1.00	1.98 (0.93–4.21)	2.03 (0.94–4.39)	1.17 (0.45–3.07)	2.39 (1.10–5.21)	0.228
**Non-haem Iron**						
Dietary intake (mg/day)	7.29	8.50	9.14	9.88	11.04	
Crude	1.00	0.98 (0.42–2.26)	1.00 (0.48–2.08)	0.89 (0.49–1.64)	0.98 (0.47–1.64)	0.856
Model adjusted ^a^	1.00	1.02 (0.40–2.64)	1.35 (0.53–3.44)	0.87 (0.44–1.71)	1.05 (0.44–2.48)	0.945
**Total Iron**						
Dietary intake (mg/day)	8.29	9.37	10.14	10.87	11.97	
Crude	1.00	0.94 (0.42–2.08)	1.21 (0.61–2.39)	0.57 (0.32–0.99)	1.18 (0.55–2.41)	0.947
Model adjusted ^a^	1.00	0.83 (0.36–2.70)	1.34 (0.63–2.84)	0.52 (0.26–1.04)	1.14 (0.54–2.40)	0.891

^a^ Adjusted for physical activity, gender, alcohol consumption, household per capita income, body mass index (BMI), high-sensitivity C-reactive protein, age, smoking status, race, total energy intake, misreporting, saturated fat and vitamin C intakes. Haem and non-haem iron were mutually adjusted.

**Table 2:** Association between quintiles of iron intake (haem, non-haem and total) and components of metabolic syndrome in ISA-Capital. São Paulo, Brazil, 2008.

Models	Quintiles of Iron Intake by Class, Odds Ratio (95% Confidence Interval)					*p* for Trend
	Q1	Q2	Q3	Q4	Q5	
Hypertension or Hypertensive Drug Therapy for Hypertension						
**Haem Iron**						
Crude	1.00	1.63 (0.90–2.96)	1.27 (0.60–2.71)	1.55 (0.74–3.23)	1.51 (0.78–2.93)	0.345
Model adjusted ^a^	1.00	1.72 (0.77–3.82)	1.39 (0.60–3.21)	2.14 (0.97–4.72)	1.59 (0.73–3.49)	0.183
**Non-Haem Iron**						
Crude	1.00	0.96 (0.51–1.77)	0.78 (0.39–1.57)	0.99 (0.51–1.93)	0.54 (0.24–1.26)	0.226
Model adjusted ^b^	1.00	0.73 (0.33–1.63)	0.65 (0.28–1.51)	0.78 (0.34–1.81)	0.40 (0.15–1.10)	0.135
**Total Iron**						
Crude	1.00	1.21 (0.71–2.05)	1.14 (0.55–2.39)	0.66 (0.31–1.39)	0.79 (0.37–1.68)	0.279
Model adjusted ^c^	1.00	0.85 (0.43–1.66)	0.93 (0.40–2.16)	0.51 (0.21–1.29)	0.57 (0.24–1.39)	0.154
Hyperglycaemia or Therapy for Elevated Glucose						
**Haem Iron**						
Crude	1.00	1.49 (0.69–3.22)	1.94 (0.76–4.99)	0.61 (0.23–1.59)	1.84 (0.75–4.54)	0.660
Model adjusted ^a^	1.00	1.56 (0.56–4.34)	2.53 (0.98–6.57)	0.66 (0.18–2.33)	1.65 (0.63–4.33)	0.746
**Non-Haem Iron**						
Crude	1.00	2.87 (0.96–8.57)	1.78 (0.56–5.63)	2.86 (1.19–6.89)	2.87 (0.99–8.36)	0.070
Model adjusted ^b^	1.00	3.21 (1.11–9.27)	1.93 (0.60–6.26)	2.92 (1.10–7.72)	2.56 (0.83–7.93)	0.183
**Total Iron**						
Crude	1.00	3.64 (1.27–10.44)	1.89 (0.64–5.57)	1.86 (0.76–4.50)	4.59 (1.71–12.29)	0.036
Model adjusted ^c^	1.00	3.50 (1.28–9.61)	1.75 (0.56–5.43)	1.49 (0.54–4.11)	3.98 (1.21–13.12)	0.131
Elevated Triglycerides or Therapy for Elevated Triglycerides						
**Haem Iron**						
Crude	1.00	2.04 (0.92–4.52)	1.46 (0.81–2.63)	1.42 (0.70–2.90)	2.73 (1.25–5.96)	0.060
Model adjusted ^a^	1.00	2.75 (1.13–6.73)	1.87 (0.95–3.66)	1.64 (0.77–3.50)	2.51 (1.06–5.91)	0.139
**Non-Haem Iron**						
Crude	1.00	1.47 (0.77–2.81)	1.54 (0.79–3.01)	1.17 (0.63–2.15)	1.16 (0.52–2.58)	0.852
Model adjusted ^b^	1.00	1.51 (0.71–3.18)	1.78 (0.85–3.72)	0.94 (0.44–2.03)	0.88 (0.31–2.47)	0.554
**Total Iron**						
Crude	1.00	1.11 (0.51–2.43)	1.70 (0.88–3.27)	1.03 (0.54–2.00)	1.19 (0.55–2.56)	0.700
Model adjusted ^c^	1.00	1.22 (0.54–2.76)	1.95 (1.00–3.86)	0.97 (0.43–2.18)	0.99 (0.43–2.28)	0.821
Reduced HDL-c (high-density lipoprotein cholesterol)						
**Haem Iron**						
Crude	1.00	1.27 (0.64–2.52)	0.88 (0.42–1.85)	0.81 (0.33–1.95)	1.70 (0.82–3.50)	0.506
Model adjusted ^a^	1.00	1.22 (0.56–2.66)	1.15 (0.46–2.87)	1.06 (0.41–2.78)	2.09 (0.88–4.94)	0.189
**Non-Haem Iron**						
Crude	1.00	0.71 (0.30–1.66)	0.73 (0.38–1.39)	0.89 (0.42–1.89)	1.26 (0.65–2.47)	0.477
Model adjusted ^b^	1.00	0.68 (0.30–1.54)	0.67 (0.37–1.21)	0.87 (0.36–2.2)	1.16 (0.53–2.55)	0.641
**Total Iron**						
Crude	1.00	0.55 (0.25–1.19)	0.68 (0.37–1.27)	0.65 (0.32–1.33)	1.21 (0.61–2.41)	0.604
Model adjusted ^c^	1.00	0.50 (0.24–1.06)	0.58 (0.31–1.09)	0.57 (0.25–1.32)	1.16 (0.51–2.63)	0.746
Increased Waist Circumference						
**Haem Iron**						
Crude	1.00	1.72 (0.62–4.79)	0.99 (0.50–1.98)	0.85 (0.35–2.07)	1.14 (0.50–2.61)	0.650
Model adjusted ^d^	1.00	1.61 (0.56–4.62)	1.18 (0.55–2.53)	0.95 (0.36–2.48)	0.99 (0.39–2.54)	0.644
**Non-Haem Iron**						
Crude	1.00	1.51 (0.67–3.41)	1.04 (0.56–1.93)	1.39 (0.79–2.45)	1.29 (0.56–2.97)	0.555
Model adjusted ^e^	1.00	1.38 (0.48–3.93)	1.03 (0.43–2.49)	1.39 (0.68–2.83)	1.23 (0.47–3.20)	0.659
**Total Iron**						
Crude	1.00	1.42 (0.62–3.24)	0.94 (0.49–1.78)	1.31 (0.71–2.40)	1.42 (0.63–3.20)	0.426
Model adjusted ^f^	1.00	0.97 (0.38–2.49)	0.72 (0.30–1.73)	1.25 (0.58–2.71)	1.00 (0.39–2.56)	0.837

^a^ Adjusted for physical activity, gender, alcohol consumption, household per capita income, BMI, high-sensitivity C-reactive protein, age, smoking status, race, total energy intake, misreporting, saturated fat, vitamin C and nonhaem iron intakes. ^b^ Adjusted for physical activity, gender, alcohol consumption, household per capita income, BMI, high-sensitivity C-reactive protein, age, smoking status, race, total energy intake, misreporting, saturated fat, fibre, vitamin C and haem iron intakes. ^c^ Adjusted for physical activity, gender, alcohol consumption, household per capita income, BMI, high-sensitivity C-reactive protein, age, smoking status, race, total energy intake, misreporting, saturated fat, fibre, vitamin C intakes. ^d^ Adjusted for physical activity, gender, alcohol consumption, household per capita income, high-sensitivity C-reactive protein, age, smoking status, race, total energy intake, misreporting, saturated fat, vitamin C and non-haem iron intakes. ^e^ Adjusted for physical activity, gender, alcohol consumption, household per capita income, high-sensitivity C-reactive protein, age, smoking status, race, total energy intake, misreporting, saturated fat, fibre, vitamin C and haem iron intakes. ^f^ Adjusted for physical activity, gender, alcohol consumption, household per capita income, high-sensitivity C-reactive protein, age, smoking status, race, total energy intake, misreporting, saturated fat, fibre and vitamin C intakes.
